# Supplementary material for: miRNAs as Regulators of the Early Local Response to Burn Injuries
Source: Int J Mol Sci. 2021 Aug 26;22(17):9209. doi: 10.3390/ijms22179209 (PMC8430593; doi:10.3390/ijms22179209)
Supplement: Supplementary file 1 [file ijms-22-09209-s001.zip › Supplementary Materials.pdf]

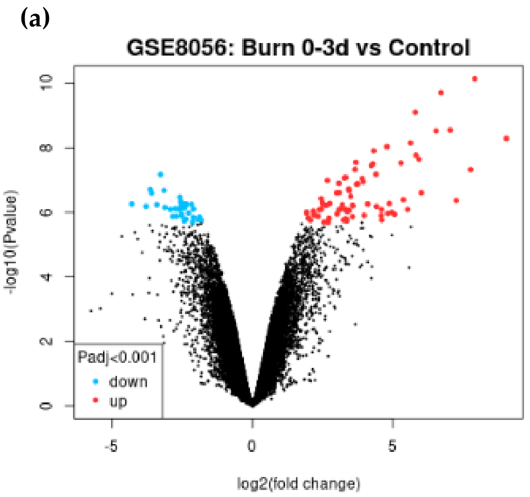

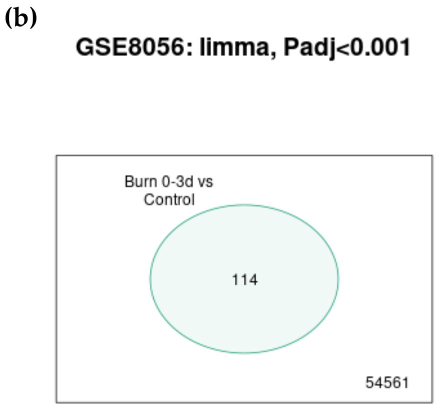

Figure S1.

(a) Volcano Plot, (b) Venn diagram

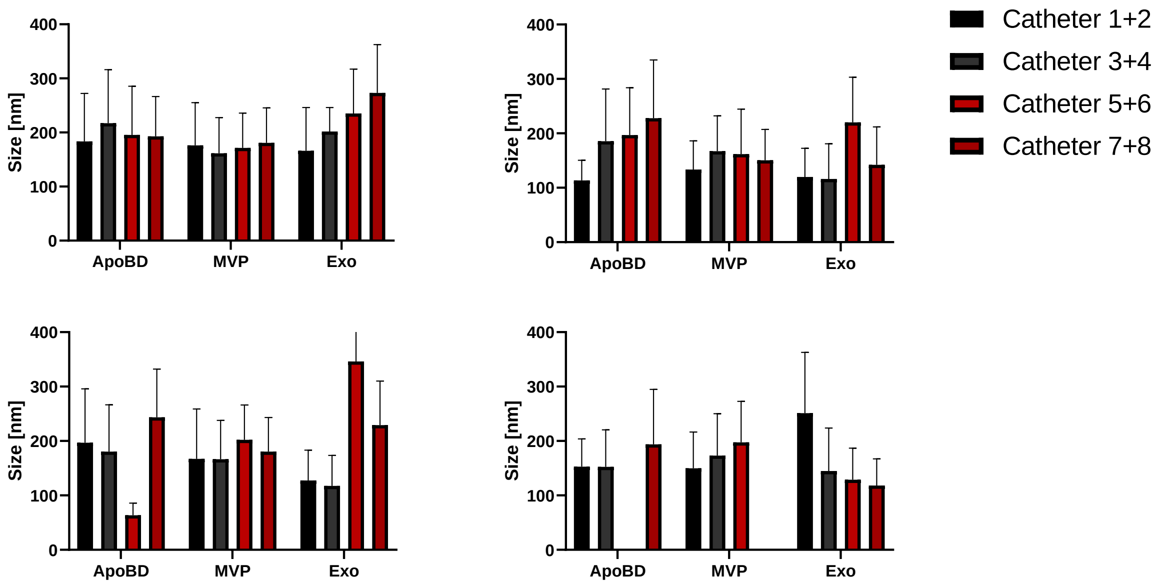

Figure S2. Size distribution of particles in dISF of an ex vivo skin model for burn injuries. Single experiments depicted seperately.

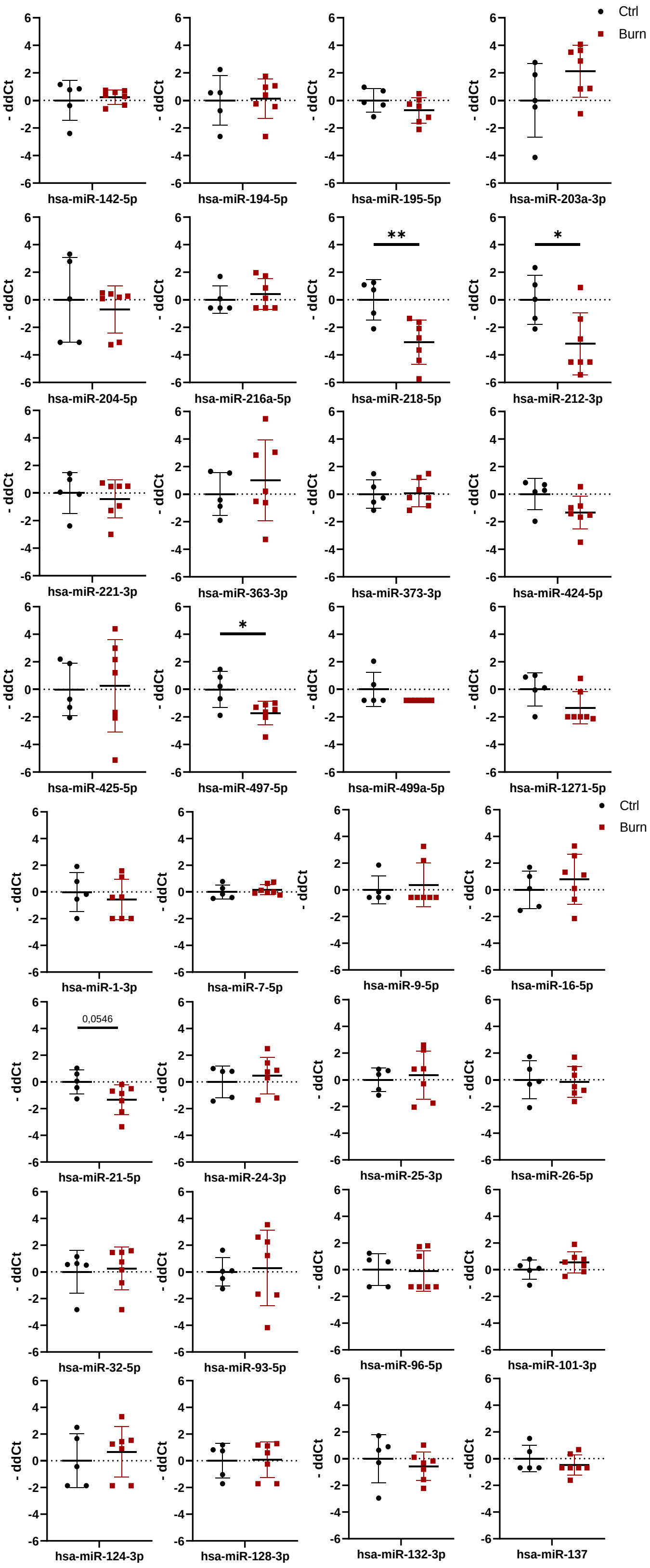

Figure S3. Characterization of miRNAs in dISF in an ex vivo human skin model for burn injuries – all analysed miRNAs. Data are derived from 3 independent experiments and presented as individual values of -ddCt, normalized to an interplate calibrator and expressed relative to controls, with means (line) and standard deviation (whiskers). Significance was tested with T-test, p-values <0.05 were considered as statistically significant, with *, **, *** indicating p<0.05, p<0.01, p<0.001.

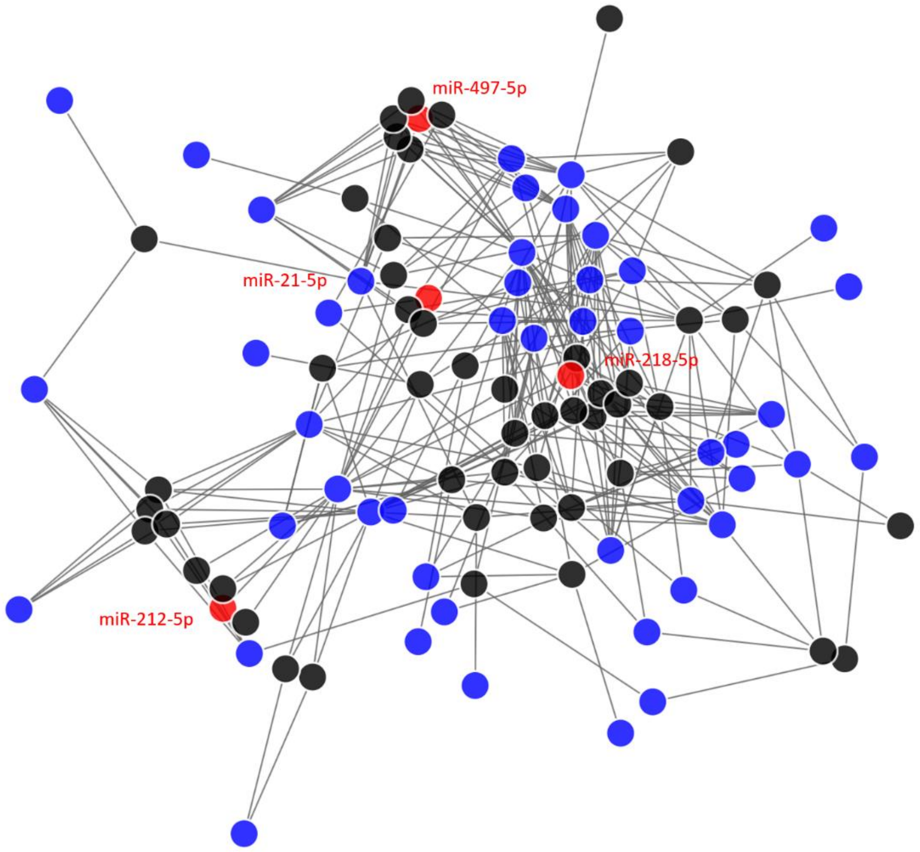

Figure S4. Interaction network of miRNAs (black and red) with the selected mRNA target genes (blue). The miRNAs found to be differentially regulated are displayed in red. There is an interactive network available with a mouse-over feature.

Table S1. miRNAs with the number of interactions based on 2 different tools that were considered as putative early burn response regulators. miRNAs for analysis have been selected by number of interactions with the 114 selected gene targets. Those miRNAs were selected that 1) preferably were found by 2 methods of screening (TargetScan Human conserved interactions among mammals) and miRWalk miRNA targets.

MiRNAs putative targeting the 114 gene targets	TargetScan Human N° of targets	Overlapping with miRWalk miRNA Targets
miR-1-3p	8	1
miR-363-3p	7	1
miR-124-3p	6	1
miR-16-5p	6	1
miR-497-5p	6	1
miR-21-5p	5	1
miR-128-3p	5	1
miR-24-3p	4	1
miR-194-5p	3	1
miR-221-3p	3	1
miR-93-5p	3	1
miR-212-3p	2	1
miR-302c-3p	2	1
miR-425-5p	2	1
miR-204-5p	2	1
miR-132-3p	2	1
miR-7-5p	1	1
miR-216a-5p	1	1
miR-203a-3p	17	0
miR-181-5p	11	0
miR-101-3p	10	0
miR-19-3p	9	0
miR-23-3p	9	0
miR-499a-5p	8	0
miR-142-5p	7	0
miR-200bc-3p	7	0
miR-25-3p	7	0
miR-27-3p	7	0
miR-32-5p	7	0
miR-367-3p	7	0
miR-520-3p	7	0
miR-92-3p	7	0
miR-9-5p	7	0
miR-1271-5p	6	0
miR-137	6	0
miR-15-5p	6	0
miR-182-5p	6	0
miR-183-5p	6	0
miR-195-5p	6	0
miR-218-5p	6	0
miR-26-5p	6	0
miR-424-5p	6	0
miR-96-5p	6	0
miR-29-3p	5	0
miR-302-3p	5	0
miR-30-5p	5	0
miR-372-3p	5	0
miR-373-3p	5	0

Table S2. Selected miRNAs for gene expression analysis.

miRBase ID	miRBase Accession	Mature miRNA sequence	GeneGlobe ID
hsa-miR-1-3p	MIMAT0022838	5'AUAUACAGGGGGAGACUCUUAU	YP02103158
hsa-miR-363-3p	MIMAT0000707	5'AAUUGCACGGUAUCCAUCUGUA	YP00204726
hsa-miR-124-3p	MIMAT0000422	5'UAAGGCACGCGGUGAAUGCC	YP00206026
hsa-miR-16-5p	MIMAT0000069	5'UAGCAGCACGUAAAUAUUGGCG	YP00205702
hsa-miR-497-5p	MIMAT0002820	5'CAGCAGCACACUGUGGUUUGU	YP00204354
hsa-miR-21-5p	MIMAT0000076	5'UAGCUUAUCAGACUGAUGUUGA	YP00204230
hsa-miR-128-3p	MIMAT0000424	5'UCACAGUGAACCGGUCUCUUU	YP00205995
hsa-miR-24-3p	MIMAT0000080	5'UGGCUCAGUUCAGCAGGAACAG	YP00204260
hsa-miR-194-5p	MIMAT0000460	5'UGUAACAGCAACUCCAUGUGGA	YP00204080
hsa-miR-221-3p	MIMAT0000278	5'AGCUACAUUGUCUGCUGGGUUUC	YP00204532
hsa-miR-93-5p	MIMAT0000093	5'CAAAGUGCUGUUCGUGCAGGUAG	YP00204715
hsa-miR-212-3p	MIMAT0000269	5'UAACAGUCUCCAGUCACGGCC	YP00204170
hsa-miR-302c-3p	MIMAT0000717	5'UAAGUGCUUCCAUGUUUCAGUGG	YP00204403
hsa-miR-425-5p	MIMAT0003393	5'AAUGACACGAUCACUCCCGUUGA	YP00204337
hsa-miR-204-5p	MIMAT0000265	5'UUCCCUUUGUCAUCCUAUGCCU	YP00206072
hsa-miR-132-3p	MIMAT0000426	5'UAACAGUCUACAGCCAUGGUCG	YP00206035
hsa-miR-7-5p	MIMAT0000252	5'UGGAAGACUAGUGAUUUUGUUGU	YP00205877
hsa-miR-216a-5p	MIMAT0000273	5'UAAUCUCAGCUGGCAACUGUGA	YP00204167
hsa-miR-203a-3p	MIMAT0000264	5'GUGAAAUGUUUAGGACCACUAG	YP00205914
hsa-miR-101-3p	MIMAT0000099	5'UACAGUACUGUGAUAACUGAA	YP00204786
hsa-miR-499a-5p	MIMAT0002870	5'UUAAGACUUGCAGUGAUGUUU	YP00205935
hsa-miR-142-5p	MIMAT0000433	5'CAUAAAGUAGAAAGCACUACU	YP00204722
hsa-miR-25-3p	MIMAT0000081	5'CAUUGCACUUGUCUCGGUCUGA	YP00204361
hsa-miR-32-5p	MIMAT0000090	5'UAUUGCACAUUACUAAGUUGCA	YP00204792
hsa-miR-367-3p	MIMAT0000719	5'AAUUGCACUUUAGCAAUGGUGA	YP00204784
hsa-miR-9-5p	MIMAT0000441	5'UCUUUGGUUAUCUAGCUGUAUGA	YP00204513
hsa-miR-1271-5p	MIMAT0005796	5'CUUGGCACCUAGCAAGCACUCA	YP00204351
hsa-miR-137	MIMAT0000429	5'UUAUUGCUUAAGAAUACGCGUAG	YP00206062
hsa-miR-182-5p	MIMAT0000259	5'UUUGGCAAUGGUAGAACUCACACU	YP00206070
hsa-miR-183-5p	MIMAT0000261	5'UAUGGCACUGGUAGAAUUCACU	YP00206030
hsa-miR-195-5p	MIMAT0000461	5'UAGCAGCACAGAAAUAUUGGC	YP00205869
hsa-miR-218-5p	MIMAT0000275	5'UUGUGCUUGAUCUAACCAUGU	YP00206034
hsa-miR-26-5p	MIMAT0000082	5'UUCAAGUAAUCCAGGAUAGGCU	YP00206023
hsa-miR-424-5p	MIMAT0001341	5'CAGCAGCAAUUCAUGUUUUGAA	YP00204736
hsa-miR-96-5p	MIMAT0000095	5'UUUGGCACUAGCACAUUUUUGCU	YP00204417
hsa-miR-372-3p	MIMAT0000724	5'AAAGUGCUGCGACAUUUGAGCG	YP00204137
hsa-miR-373-3p	MIMAT0000726	5'GAAGUGCUUCGAUUUUGGGGUGU	YP00204604

Table S3. TaqMan assays used for gene expression analysis with qPCR.

TaqMan Gene expresison assay	Assay ID
TaqMan Assays (WNT2B)	Hs00921614_m1
TaqMan Assays (TIMP3)	Hs00165949_m1
TaqMan Assays (PI15)	Hs00210658_m1
TaqMan Assays (SOX5)	Hs01552788_g1
TaqMan Assays (MME)	Hs00153510_m1
TaqMan Assays (DKK2)	Hs00205294_m1
TaqMan Assays (TNC)	Hs01115665_m1
TaqMan Assays (LIPG)	Hs00195812_m1
TaqMan Assays (MT1G)	Hs04401199_s1
TaqMan Assays (DDX3Y)	Hs00965254_gH
TaqMan Assays (SLC2A3)	Hs00359840_m1
TaqMan Assays (TBP)	Hs00427620_m1
TaqMan Assays (RPLP0)	Hs00420895_gH
TaqMan Assays (GAPDH)	Hs02786624_g1
